# The global burden of sore throat and group A *Streptococcus* pharyngitis: A systematic review and meta-analysis

**DOI:** 10.1016/j.eclinm.2022.101458

**Published:** 2022-05-20

**Authors:** Kate M. Miller, Jonathan R. Carapetis, Chris A. Van Beneden, Daniel Cadarette, Jessica N. Daw, Hannah C. Moore, David E. Bloom, Jeffrey W. Cannon

**Affiliations:** a Wesfarmers Centre of Vaccines and Telethon Kids Institute, University of Western Australia, PO Box 855, West Perth, Nedlands, WA 6872, Australia; b Perth Children's Hospital, Nedlands, WA, Australia; c CDC Foundation, Atlanta, GA, USA; d Department of Global Health and Population, Harvard T.H. Chan School of Public Health, Boston, MA, USA

**Keywords:** Pharyngitis, Sore throat, Streptococcus, Incidence, Surveillance, Burden

## Abstract

**Background:**

Contemporary data for the global burden of sore throat and group A *Streptococcus* (Strep A) pharyngitis are required to understand the frequency of disease and develop value propositions for Strep A vaccines.

**Methods:**

We used Clarivate Analytics’ Web of Science platform to search WoS core collection, PubMed, Medline, data citation index, KCI-Korean Journal Database, Russian Science Citation Index, and the SciELO Citation Index for articles published between Jan 1, 2000, and Feb 15, 2021, from any country and in any language. The risk of bias was assessed using the JBI critical appraisal checklist. We used random-effects meta-analyses to pool sore throat and Strep A sore throat incidence rates from community-based studies. Our study was registered with PROSPERO (CRD42020181103).

**Findings:**

Of 5,529 articles identified by the search strategy, 26 studies met the inclusion criteria, but only two included data to determine incidence among adults. The pooled incidence rate, calculated for children only, was 82.2 episodes per 100 child-years (95% CI 25.2–286.3, I^2^ = 100%) for sore throat (7 studies; 7,964 person years) and 22.1 episodes per 100 child-years (95% CI 14.7–33.1, I^2^ = 98%) for Strep A sore throat (9 studies; 15,696 person years). The pooled cumulative incidence rate of sore throat from five studies was 31.9 per 100 children. There was significant methodological and statistical heterogeneity among studies, and five of 26 studies had a risk of bias score less than five (range: nine [maximum score] to one).

**Interpretation:**

Strep A sore throat has a considerable global burden. However, methodologically standardised studies are required to quantify that burden, analyse differences in rates between populations, and evaluate the likely impact of future Strep A vaccines.

**Funding:**

This study was funded by Wellcome Trust 215,490/Z/19/Z.

**Research in context**

**Evidence before this study**

The literature search for this review was performed using Clarivate Analytics’ Web of Science from 2000 to February 15, 2021, with no language restrictions. Search terms included “sore throat” OR pharyngitis OR tonsillitis OR tonsillopharyngitis OR “throat infection” or “*Streptococcus* A” AND incidence OR prevalence OR burden OR DALYs OR epidemiology AND global OR worldwide. We included studies that estimated the incidence or DALYs of sore throat or Strep A sore throat.

**Added value of this study**

We present contemporary estimates for the global burden of Strep A sore throat and first-ever estimates of associated DALYs. We estimated that 288.6 million episodes of Strep A sore throat occur among children (5–14 years) each year globally, accounting for more than 100,000 DALYs. We further estimated that one in three children experience one or more episodes of sore throat over a 12-month period.

**Implications of the available evidence**

The World Health Organization cites Strep A sore throat as one of several preferred clinical indications for vaccines. The DALYs that Strep A sore throat causes in children contribute substantially to the global burden of Strep A. In addition to reducing the burden of downstream complications stemming from Strep A sore throat, including RHD, the implementation of a Strep A vaccine could reduce antibiotic use for sore throat.

Alt-text: Unlabelled box

## Introduction

Upper respiratory infection by group A *Streptococcus* (Strep A) and other pathogens can result in a sore throat and clinical diagnosis of pharyngitis or tonsillitis. Strep A sore throat (and tonsillitis) can progress to more severe infection, such as peritonsillar abscess or invasive infections; Strep A sore throat can also result in post-infection sequelae including acute post-streptococcal glomerulonephritis, acute rheumatic fever (ARF), and rheumatic heart disease (RHD). There is limited contemporary data for the global burden of all Strep A related diseases. Based on 2010 estimates that each year RHD caused more than 345,000 deaths^1^ added to previous estimates that Strep A invasive infection caused more than 163,000 deaths,^2^ Strep A was postulated to be the fifth most lethal pathogen in the world.^1^^,^^3^ Strep A vaccines could significantly reduce this disease burden. Moreover, the World Health Organization highlights the potential role of Strep A vaccines in reducing antibiotic treatment of presumed Strep A sore throat,^4^ which could plausibly lead to reduced levels of antibiotic resistance and associated harms.

Despite the high global burden of Strep A diseases and the existence of several promising candidate vaccines in early development, investment in late-stage development of these candidates has not been forthcoming.^5^ Therefore, formal assessment of the value of prospective Strep A vaccines from both a commercial and societal perspective is warranted. The incidence and burden of sore throat and Strep A sore throat are important inputs for assessing the value of Strep A vaccines.

A review of the burden of Strep A diseases published in 2005 estimated that more than 616 million cases of Strep A sore throat occur globally each year.^2^ Since then, a single systematic review and meta-analysis of the incidence of sore throat and Strep A sore throat has been published, which reported a pooled Strep A sore throat incidence of 10.8 episodes per 100 child-years from six studies.^6^ Collectively, these published reviews had significant limitations. The first review was based on only four studies, the most recent of which was published nearly two decades ago, and the second review only examined studies among populations considered at risk of ARF (children from low- and middle-income countries [LMICs] or Indigenous children living in high-income countries [HICs]). Consequently, there are no contemporary estimates of the global incidence or burden of sore throat and Strep A sore throat.

We aimed to update estimates of the global incidence of sore throat and Strep A sore throat and calculate the numbers of episodes and disability-adjusted life-years (DALYs) for Strep A sore throat based on the global population of 2020.

## Methods

### Search strategy and selection criteria

To inform investment cases regarding the full value of Strep A vaccination, we conducted a systematic review and meta-analysis on the global burden of Strep A sore throat. We prospectively submitted the systematic review protocol for registration with PROSPERO (CRD42020181103) and reported according to PRISMA guidelines.^7^ The study protocol is available online https://www.crd.york.ac.uk/prospero/display_record.php?recordid=181103.

We used Clarivate Analytics’ Web of Science search platform, which includes multiple databases such as WoS core collection, PubMed, Medline, data citation index, KCI-Korean Journal Database, Russian Science Citation Index, and the SciELO Citation Index to perform a literature search of papers published between 2000 and 2019 inclusive on April 13, 2020, and we conducted an updated search on February 15, 2021, to include all papers published since the original end date. The search included the following terms: tonsillopharyngitis, pharyngitis, tonsillitis, sore throat, or throat infection and epidemiology, incidence, frequency, rate, surveillance, prevalence, or burden. To supplement database searches, we used Google Scholar; using the same search terms to capture any additional studies within the gray literature, including conference proceedings. Appendix 1 provides a list of search strings. We also reviewed reference lists of each eligible paper to identify additional articles for inclusion.

Studies were eligible for inclusion if (1) incidence rates or cumulative incidence could be calculated for sore throat or Strep A sore throat (both outcomes including pharyngitis, tonsillitis, or tonsillopharyngitis) and (2) participants were recruited from settings that captured a representative sample of the general population. Studies were excluded for the following reasons: the population denominator could not be established; incidence rates were modelled, rather than observed; episodes of sore throat were limited to those caused solely by a pathogen other than Strep A (e.g., studies of only episodes of group C or G *Streptococcus* pharyngitis); or episodes of sore throat that were the result of endotracheal intubation. We also excluded studies based on healthcare presentations for sore throat as they are biased toward people accessing care and, therefore, are not representative of all people who experience a sore throat in a population. No restrictions were placed on age or language. Google translate was used to translate non-English publications.

We imported references into EndNote x8 for Windows. Two reviewers (KM and JWC) independently reviewed the titles and abstracts of papers identified by the search to select eligible studies. Papers for which a title and abstract provided insufficient detail were included in the full text review to determine eligibility for inclusion. The same two authors independently assessed all full text articles using predefined selection criteria. Discrepancies were resolved by consensus.

The two reviewers (KM and JWC) independently extracted data. Extracted data included article identification (surname of the first author and year of publication), surveillance/follow-up time, country, study design, study population, study setting, number of participants, age range, race, sampling method, case detection method, screening method and frequency, diagnostic testing method and frequency, number of people or episodes of sore throat, and number of people or episodes of Strep A sore throat. Authors of eligible studies were contacted if additional data were required.

As this review was based on community studies, we classified sore throat, pharyngitis, and tonsillopharyngitis episodes as “sore throat.” Tonsillitis episodes were considered separately and not included in meta-analysis. A case of Strep A sore throat was defined as an episode of sore throat in a person with microbiological confirmation of Strep A in the oropharynx by a positive throat culture, Rapid Antigen Detection Test (RADT), or Nucleic Acid Amplification Test.^8^ Further definitions used in this review are provided in Appendix 2.

### Quality assessment

Two authors (KM and JD) independently assessed the risk of bias for each included study using the Joanna Briggs Institute (JBI) “Checklist for Prevalence Studies.”^9^ The checklist is composed of nine questions related to study selection, measurement, and comparability of studies. Differences in scoring were resolved through discussion.

### Data analysis

Initial review of the included studies indicated a high degree of methodological heterogeneity. To quantitatively analyse studies with broadly similar study designs, we included studies with more than six months of follow-up time in random-effects meta-analyses to calculate pooled annual incidence rates (IRs) for sore throat and Strep A sore throat. Studies with six months or less of follow-up time were excluded from meta-analysis due to the seasonal nature of upper respiratory tract infections, which may affect the pooled incidence rate. Due to the limited number of studies including adults (*n* = 2), meta-analysis was limited to children aged 5–14 years. We also used random-effects meta-analyses to calculate a pooled cumulative incidence for sore throat among children. Results were displayed in Forest plots, and the I^2^ statistic quantified between-study heterogeneity. We explored potential sources of heterogeneity among studies by including the following variables in univariate meta-regression analysis: country, country income level (LMICs or HICs),^10^ follow-up frequency, and microbiological testing frequency. We also presented pooled IRs by country income level. Statistical analyses were performed using the program R (version 3.6.1) with the meta package.^11^ A narrative review was provided of all studies not included in meta-analysis.

To estimate the total number of Strep A sore throat episodes in 2020, we multiplied the pooled IRs by the 2020 global population.^12^ We then multiplied the numbers of episodes for each outcome by the weighted disability weight for upper respiratory infection (0.026) for a duration of 5 days to estimate DALYs due to Strep A sore throat.^13^

### Role of the funding source

The funder of the study had no role in the study design, data collection, data analysis, data interpretation, or writing of the report. All authors had full access to all the data in the study and had final responsibility for the decision to submit for publication.

## Results

**Figure 1:**
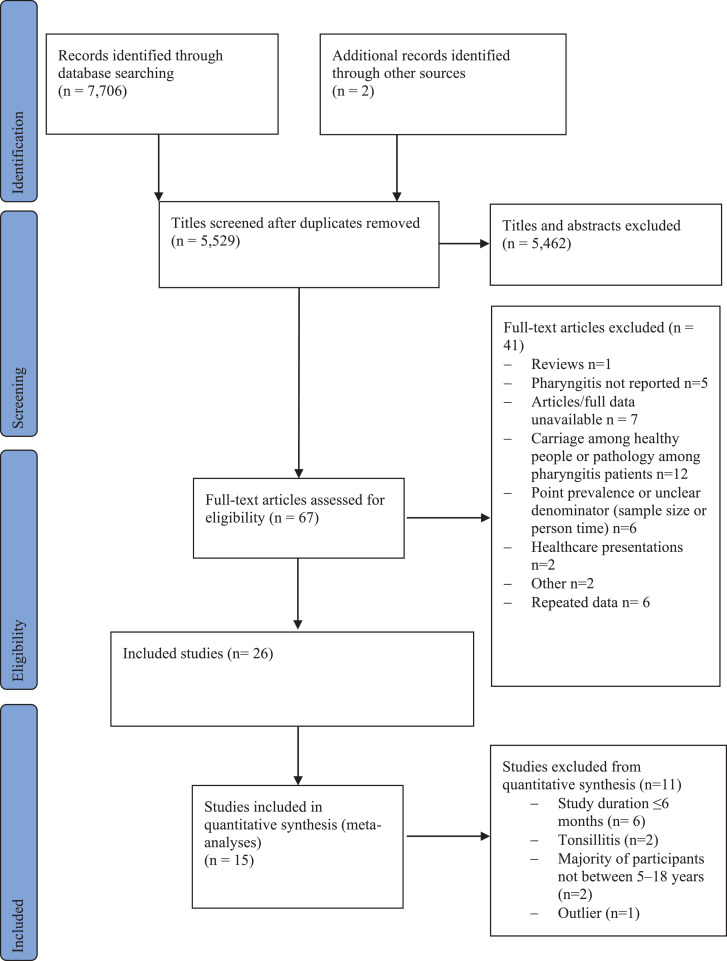
PRISMA flowchart.

The search strategy identified 5,529 articles after removal of 2,179 duplicates. Of those, 67 studies underwent full-text review and 24 met the inclusion criteria for further analysis. A further two studies were included after searching references. Thus, 26 studies met the inclusion criteria (Figure 1), of which 15 studies qualified for one or more meta-analyses (i.e., reported one or more outcomes or incidence measures), and the remaining 11 articles were included in the narrative synthesis.

Using JBI's critical appraisal checklist tool for prevalence studies, only one study attained a maximum score of nine, two studies attained a score of eight, five studies attained a score of seven, seven studies attained a score of six, six studies attained a score of five, two studies attained a score of four or three, and one study attained a score of one. Minimal risk of bias was observed for study sampling and validity of measurements for confirming cases of Strep A sore throat. The main source of potential bias was related to the assessment of coverage bias and study comparability due to insufficient detail on study participants and setting.

All eligible studies were published between 2000 and 2020 inclusive and reflected data collected between 1992 and 2017 inclusive. The recency of data was limited; only one study included data collected in the past five years. Incidence rates were obtained from 17 studies, and cumulative incidence rates were abstracted from 11 studies (Appendix 5) (two studies reported both incidence and cumulative incidence rates). Eleven of the 26 studies provided an IR directly,^14^, ^15^, ^16^, ^17^, ^18^, ^19^, ^20^, ^21^, ^22^, ^23^, ^24^, ^25^ and we calculated the IR or cumulative incidence for the remaining studies.^8^^,^^26^, ^27^, ^28^, ^29^, ^30^, ^31^, ^32^, ^33^, ^34^, ^35^, ^36^, ^37^, ^38^

**Figure 2:**
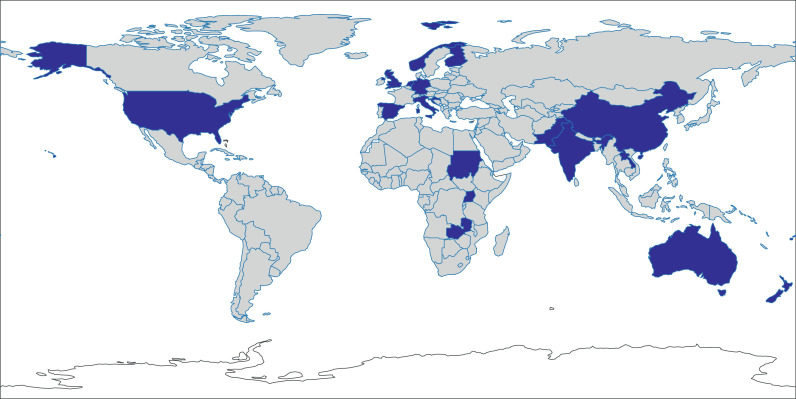
Countries in which studies described the incidence of sore throat or Strep A sore throat.

The studies were conducted in 19 countries in Africa, Asia, Europe, North America, and Oceania (Figure 2). All but one study included children.^26^ All studies included both male and female participants. The duration of the studies ranged from four weeks^8^ to five years.^32^ 18 studies had a prospective study design; the remaining eight were retrospective. More than half (58%) of the studies were based in schools, 23% were based in the community, and the remainder used household surveys (19%). The IRs from control arms of randomized control trials were used for three studies. 13 of the 17 studies reporting incidence rates involved active surveillance compared with two of the 11 studies reporting cumulative incidence. The frequency of screening for new cases ranged from daily to annually. The quality of included studies, as measured by the JBI checklist for prevalence studies, ranged from poor to excellent (minimum and maximum scores of 1 and 9) (Appendix 3).

Countries with available data are marked in blue.

### Incidence of sore throat

**Figure 3:**
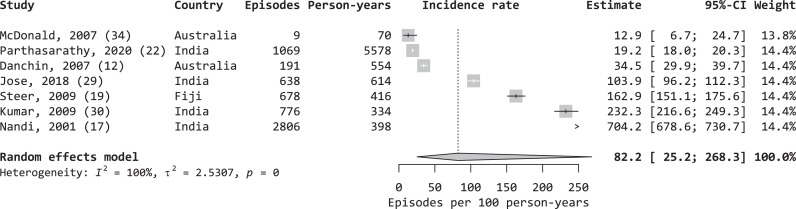
Incidence rates of sore throat among children.

12 studies reported the incidence of sore throat (Table 1) with incidence rates ranging from 0.006 episodes per 100 child-years^20^ to 1,290.2 per 100 child-years.^8^ Of these, 11 studies provided data on the incidence of sore throat among children specifically (with only two also including age-specific rates among adults), while the other provided the incidence across all ages. Due to the limited data on adults, meta-analysis was limited to children, which were most commonly aged 5–14 years. Of the 11 studies among children, seven were prospective studies with more than six months of follow up and were included for meta-analysis. The pooled IR of sore throat was 82.2 episodes per 100 child-years (95% confidence interval [CI], 25.2–286.3 per 100 child-years, I^2^ = 100%; Figure 3).

**Table 1:** Characteristics of studies with data on the incidence rate of sore throat.

Source	Study year(s)	Country	Setting	Study design	Surveillance type	Screening frequency and case identification	Age	Participants	Surveillance duration (months)	Incidence (per 100 PY)
Negi, 2018^21^	January 2015 to December 2016	India	School	Prospective (RCT)	Passive	Unclear	5–18	24,980	24	0.006
McDonald, 2006^37^	August 2003 to June 2005	Australia	Householdǂ	Prospective	Active	At least monthly culture for all participants	All	1,173	23	0.5Δ
McDonald, 2007^36^	September 2004 to September 2005	Australia	Householdǂ	Prospective	Active	At least monthly culture for all participants	All <15 ≥15	145 91 54	10 10 10	13.4Δ 12.9Δ 14.4Δ
Parthasarathy, 2020^25^	August 2012 to August 2014	India	Household	Prospective	Active	Weekly screening for sore throat by trained field workers	≤10	3,765	25	19.2
Musuku, 2017^38^	September 2014 to November 2015	Zambia	School	Retrospective	Passive	One-off self-report	5–29^	3,462	12	32.0
Danchin, 2007^15^	August 2001 to December 2002	Australia	Community	Prospective	Passive	Symptomatic presentation to clinic	1–18 >18	470 358	16 16	34.5 13.5
Kostic, 2019^39^	September 2016 to December 2016	Croatia	School	Prospective	Active	Twice monthly	6–7	81	3	46.7
Jose, 2018^31^	Not specified	India	School	Prospective	Active	Weekly physical assessment	7–11	307	24*	103.9
Steer, 2009^22^	March 2006 to November 2006	Fiji	School	Prospective	Active	Twice weekly class query	5–14	667	9	162.9
Kumar, 2009^32^	November 2000 to January 2002	India	School	Prospective	Active	Fortnightly physical assessment	5–15	334	12*	232.3
Nandi, 2001^20^	April 1995 to March 1996	India	Household	Prospective	Active	Fortnightly interview	5–15	536	12	704.2Δ
DeWyer, 2020^9^	March 2017 to April 2017	Uganda	School	Prospective	Active	Daily class query + weekly physical assessment	5–16	532	1	1290.2Δ

PY = person-years. RCT = Randomised controlled trial.

ǂ Study took place among remote Indigenous communities.

⁎ Surveillance was assumed to occur for 12 months for each school year when estimating person-time, which underestimates incidence rates in studies where surveillance duration was less than 12 months.

^ 98% <20 yrs.

Δ Differs from incidence reported in study.

A forest plot of the rates of sore throat among children (incidence) with 95% confidence intervals (CI), excluding studies with less than 6 months follow-up (random effects model, heterogeneity: I2 = 100%, *p* = 0).

We excluded four studies of children from the meta-analysis. The study by DeWyer et al. of Ugandan children aged 5–16 years provides the only data from a low-income country, but the surveillance period was limited to one month, with an extrapolated IR of 1,290.2 episodes per 100 person-years.^8^ Similarly, the Croatian study by Kostic et al. was a prospective study utilising active surveillance methodology among children (6–7 years old) over a relatively short duration (3 months),^38^ with an extrapolated IR of 46.7 episodes per 100 person-years. The study by Negi et al. among Indian children aged 6–18 years was an outlier (sore throat IR of 0.006 episodes per 100 child-years) compared with the other studies reviewed; the study authors explained that the low rate was likely due to passive surveillance and underreporting.^20^ Lastly, the Zambian study by Musuku et al. used a retrospective, rather than prospective, study design and reported an IR of 32.0 episodes per 100 person-years for individuals aged 5–29 years.^37^

Three studies included data to determine IRs among adults or all ages; all were from Australia. Based on data from two studies reported by McDonald et al., the IRs of sore throat among Indigenous people in remote Northern and Central Australian communities were 0.5 episodes per 100 person-years and 13.4 episodes per 100 person-years, respectively.^35^^,^^36^ The Central Australian study also indicated an IR of 14.4 episodes per 100 person-years among adults (≥15 years old), which was comparable to the IR (13.5 episodes per 100 person-years) among the parents of children included in the Melbourne study by Danchin et al.^14^

Five studies reported one or more episodes of sore throat in school-aged children (ranging from 5 to 29 years old) within a study period of 9–12 months. From these studies, a pooled cumulative incidence of 31.9 per 100 children (i.e., 31.9% of children) had one or more episodes of sore throat during the study periods (Appendix 4, Figure A1). Appendices 4 and 5 report further results.

### Incidence of Strep A sore throat

15 studies reported the incidence of Strep A sore throat: 14 reported incidence rates^8^^,^^14^^,^^17^^,^^19^^,^^21^^,^^25^^,^^28^, ^29^, ^30^, ^31^, ^32^, ^33^^,^^35^^,^^36^^,^^38^ (Table 2), and one reported cumulative incidence.^29^ Of the 15 studies, 12 were conducted among children, one among children and their parents, and two among participants of all ages. Episodes of Strep A sore throat were ascertained via culture from throat swabs in all but one study; the study by DeWyer et al. used RADTs for Strep A, with throat culture performed on portions of RADT positive and negative cases.^8^ A single study provided an IR for serologically confirmed Strep A sore throat.^14^ The IR of Strep A sore throat ranged from 0.12 episodes per 100 child-years^36^ to 540.0 episodes per 100 child-years.^8^

**Table 2:** Characteristics of studies with data on the incidence rate of Strep A sore throat.

Source	Study year	Country	Setting	Study design	Surveillance type	Screening frequency and case identification	Microbiological testing method and frequency	Age (years)	No. of participants	Surveillance duration (months)	Incidence (per 100 PY)
McDonald, 2006^37^	August 2003 to June 2005	Australia	Householdǂ	Prospective	Active	At least monthly physical assessment	At least monthly culture for all participants	All ages	1173	23	0.12 Δ
McDonald, 2007^36^	September 2004 to September 2005	Australia	Householdǂ	Prospective	Active	At least monthly physical assessment	At least monthly culture for all participants	All ages	145	10	0.9 Δ
Kumar, 2009^32^	November 2000 to January 2002	India	School	Prospective	Active	Fortnightly physical assessment	Culture episodes during fortnightly screening	5–15	334	12*	5.4
Danchin, 2007^15^	August 2001 to December 2002	Australia	Community	Prospective	Passive	Symptomatic presentation to clinic	Culture every episode	1–18 >18	470 358	16 16	11.9 4.7
Steer, 2009^22^	March 2006 to November 2006	Fiji	School	Prospective	Active	Twice weekly class query	Culture episodes during twice-weekly screening	5–14	667	9	14.7
Kumar, 2012^18^	July 2002 to April 2004	India	School	Prospective	Active	Weekly physical assessment	Culture episodes during weekly screening	7–11	241	21	16.6
Jose, 2018^31^	Not specified	India	School	Prospective	Active	Weekly physical assessment	Culture episodes during weekly screening	7–11	307	24*	17.9
Nandi, 2001^20^	April 1995 to March 1996	India	Household	Prospective	Active	Fortnightly interview	Culture episodes during fortnightly screening	5–15	536	12	30.9
Kostic, 2019^39^	September 2016 to December 2016	Croatia	School	Prospective	Active	Twice monthly	Culture at clinic	6–7	81	3	31.1
Lewnard, 2020^33^	October 1998 to May 2003	USA	School	Prospective	Active	Fortnightly physical assessment + symptomatic presentation to clinic	Culture every episode	3–14	145	56*	47.3
Lin, 2008^34^	September 1992 to July 1993	China	School	Prospective (RCT)	Active	Daily class query + daily parent report	Culture episodes daily during screening	9–12	193	9	49.7
Lennon, 2000^26^	Not specified	New Zealand	School	Prospective	Passive	Symptomatic presentation to clinic	Culture every episode	School children	12,572⁎⁎	12*	50.0
Di Pierro, 2016^29^	September 2015 to March 2016	Italy	School	Prospective (RCT)	Passive	Symptomatic presentation to clinic	Culture every episode	2–3	111	6	121.1
DeWyer, 2020^9^	March 2017 to April 2017	Uganda	School	Prospective	Active	Daily class query + weekly physical assessment	RADT every episode; culture ¼ RADT+ and ½ RADT-	5–16	532	1	540.0

PY = person-years. RADT = rapid antigen detection test. RCT = Randomised controlled trial.

ǂ Study took place among remote Indigenous communities.

⁎ Surveillance was assumed to occur for 12 months for each school year when estimating person-time, which underestimates incidence rates in studies where surveillance duration was less than 12 months.

⁎⁎ Estimated from reported incidence rate and number of Strep A sore throat episodes.

Δ Differs from incidence reported in study.

**Figure 4:**
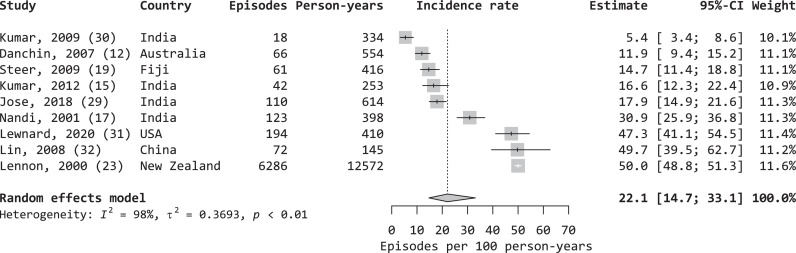
Pooled incidence rate of Strep A sore throat for children.

Nine of the 13 studies that included children were conducted prospectively over a period of nine months or more.^14^^,^^17^^,^^19^^,^^21^^,^^25^^,^^30^, ^31^, ^32^, ^33^ From those studies, the pooled IR of Strep A sore throat for children, most commonly aged 5–14 years, in those studies was 22.1 (95% CI 14.7–33.1) episodes per 100 child-years (Figure 4). Significant heterogeneity was present among those studies (*I*^2^ = 98%, *p* < 0.001), which could not be explained by country, country income level, study duration, follow-up frequency, or microbiological testing frequency. From the sub-group analysis, no statistical evidence indicated a difference in pooled IRs of Strep A sore throat between LMICs and HICs (*p* = 0.190), with respective pooled IRs of 18.6 episodes (95% CI 11.6–29.9) and 30.9 episodes (95% CI 17.1–55.6) per 100 child-years. Given 1.3 billion children aged 5–14 years of age in 2020, the estimated global burden of Strep A sore throat among children is 288.6 million episodes and 0.1 million DALYs.

Random effects model, heterogeneity: I2 = 98%, *p* < 0.01.

Four studies of children were not included in the pooled analysis. Data from the Italian study by Di Pierro et al. among 3-year-old children suggest an incidence rate of 121.1 episodes per 100 person-years based on a six-month follow-up period.^28^ The Ugandan study by DeWyer et al. among children aged 5–16 years reported an extrapolated IR of 540.0 episodes per 100 person-years from one month of surveillance.^8^ The Croatian study by Kostic et al. of children aged 6–7 years during autumn months had an extrapolated IR of 31.1 episodes per 100 person-years based on three months of follow up.^38^ Lastly, in the cumulative incidence study by Dierksen et al. among New Zealand children aged 5–13 years, 15.7% experienced one or more episodes of Strep A sore throat in a 10-month period.^29^

The studies of participants of any age were both conducted in remote Indigenous Australian communities, where the incidence of ARF is high.^35^^,^^36^ One study was conducted among three communities in the Northern Territory. The authors reported no cases of Strep A sore throat among 633 children under 15 years of age and 4.0 episodes per 100 person-years among adults. Although this study included data for children, we did not include it in the meta-analysis because the authors reported inconsistent follow-up among participants during the one-to-two-year surveillance period. The other study conducted in Central Australia reported a Strep A sore throat IR of 32.0 episodes per 100 person-years for the whole population. While cases were identified among children, neither raw numbers nor age-specific rates for children were obtainable.

In addition to reporting Strep A sore throat among children, Danchin et al. reported an IR for parents of 4.7 episodes per 100 person-years and an IR for families of 8.8 episodes per 100 person-years.^14^ Further, it was the only study to provide data on serologically confirmed Strep A sore throat, which was 5.7 episodes per 100 person-years for families.

## Discussion

This paper comprehensively reviews studies of sore throat and Strep A sore throat incidence and estimates their global burden. We found 12 and 14 studies that directly or indirectly reported the incidence rates for sore throat and Strep A sore throat, respectively. There was considerable methodological heterogeneity among the studies. For example, studies differed in combinations of duration, follow up frequency, microbiological testing frequency, participant age, and data collection (prospective vs. retrospective). Among studies conducted prospectively for six or more months among children, meta-analyses showed significant statistical heterogeneity.

The pooled IR of sore throat episodes in our study was comparable to that reported by Pearce et al. (82.2 vs. 82.5 episodes per 100 child-years), whereas our pooled IR for Strep A sore throat was more than double their estimate (22.1 vs. 10.8 episodes per 100 child-years).^6^ A major difference between the reviews is that our study included data from populations with high and low incidences of ARF, and we found the latter populations to have moderate to high rates of Strep A sore throat.^14^^,^^32^ Our data suggest that population rates of Strep A sore throat may not be accurate predictors of ARF rates and vice versa. Emerging evidence suggesting a possible association between Strep A impetigo and ARF^36^^,^^39^^,^^40^ may partially explain the lack of association between high incidence rates of Strep A sore throat and ARF. More detailed surveillance data, such as prior Strep A respiratory tract and skin infections or strain diversity, may help to better understand changes in ARF incidence over time.^41^

Our global estimate of 288.6 million episodes of Strep A sore throat among children aged 5–14 years is lower than the global estimate of 446 million episodes among children 5–14 years published in 2005.^2^ The latter estimate was based on IRs of 40 episodes per 100 child-years in LMICs and 15 episodes per 100 child-years in HICs. However, we could not establish a significant difference in IRs between LMICs and HICs from the studies included in our review.

This review has some limitations. While the objective of this study was to estimate the global burden of Strep A sore throat, the relatively small number of studies available and limited number of communities and countries represented by the current studies limit the accuracy of these estimates. We sought to provide pooled IRs for sore throat and Strep A sore throat among children; however, few studies included children under five years of age and only one provided an age-specific rate for children aged 0–5 years. Future community-based surveillance studies may consider including children aged 3–4 years to reflect changes in early childcare centre attendance and younger age of mandated school entry in western countries (3–4 years), which through increased opportunities for exposure, are thought to be shifting the age of first acquisition earlier.

Additionally, Strep A sore throat was defined in all but one study as sore throat, or pharyngitis, with the presence of Strep A in the pharynx based on positive culture or RADT. As neither diagnostic method distinguishes between throat carriage and active infection, it is unlikely that all reported episodes were caused by Strep A. Danchin et al. demonstrated this, finding evidence of active Strep A infection (defined as a significant serological response) in only two-thirds of culture-positive episodes from children.^14^

We excluded studies based on healthcare presentations for sore throat from this review. In the USA, the rate of ambulatory care presentations in children aged 0–19 years for pharyngitis (2010–2011) was 16.2 presentations per 100 person-years,^42^ whereas community surveillance among children in the USA for Strep A pharyngitis alone was almost three times higher at 47 episodes per 100 child-years.^32^ In a survey of 15,788 individuals aged 14 years and over, Hannaford et al. found that only 38% of those who reported severe sore throat or tonsillitis presented to health services at the time of acute infection.^15^ These studies highlight the value of community surveillance to estimate the disease burden of sore throat and Strep A sore throat. Use of outpatient data is limited to the small proportion of people who present to health services for what is often considered a self-limiting disease^43^ and is further limited by diagnostic testing practices, and coding of electronic medical records. As such, outpatient data provide an incomplete estimate that is biased toward more severe cases.

Statistical and methodological heterogeneity was high among the studies, with no two studies sharing the same combination of age range, surveillance type, screening methodology, and microbiological testing protocol, which makes within- and between-country comparisons unreliable. We excluded studies with six or fewer months of follow up from meta-analysis, as the extrapolated annual rate could be biased due to the seasonal pattern of Strep A sore throat.^44^ Further, few studies included data collected within the past several years, which introduces uncertainty regarding the accuracy of disease burden estimates for 2020. For example, it is possible that there have been temporal changes in incidence, which is not reflected in our estimate. Considering prospective vaccines targeted at Strep A sore throat, more globally representative data and more consistent surveillance methodology needs to be adopted to better determine the full potential health, economic, and societal value of vaccines. This requires epidemiological protocols that include consensus case definitions and best practice surveillance guidelines to improve the accuracy of global disease estimates and allow accurate within- and between-country comparisons in the future.

Notwithstanding these limitations, our results indicate that Strep A sore throat causes 0.1 million DALYs among children each year globally. RHD, one of the gravest clinical endpoints of Strep A infection, was reportedly responsible for 0.5 million DALYs among 5–14 year-old children and 10.7 million DALYs among all age groups in 2019.^45^ Global DALYs due to other Strep A clinical manifestations, such as cellulitis, sepsis, necrotizing fasciitis, ARF, and post-streptococcal glomerulonephritis have not been estimated. This study suggests that estimating the DALYs caused by all Strep A clinical manifestations could significantly increase our understanding of the burden of Strep A and provide evidence for the importance of Strep A sore throat as a clinical indicator for vaccines.

## Contributors

KM and JWC conducted the search for articles. KM wrote the narrative review, while JWC did the statistical analysis. KM and JWC wrote the first draft of the report. JRC, CvB, HM, JD, DC, and DB reviewed the draft report and provided input to the final manuscript. KM and JWC accessed and verified the data. All authors had full access to all the data in the study and had final responsibility for the decision to submit for publication.

## Data sharing

All relevant data are within the paper and Appendices.

## Funding

This study was funded by Wellcome Trust 215,490/Z/19/Z.

## Declaration of interests

We declare no competing interests.

## Appendix Supplementary materials

Image, application 1
