# Supplementary material for: The global burden of sore throat and group A Streptococcus pharyngitis: A systematic review and meta-analysis
Source: eClinicalMedicine. 2022 May 20;48:101458. doi: 10.1016/j.eclinm.2022.101458 (PMC9124702; doi:10.1016/j.eclinm.2022.101458)
Supplement: Supplementary file 1 [file mmc1.pdf]

APPENDIX 1: SEARCH STRINGS

Topic Search (TS)=(tonsillopharyngitis OR pharyngitis OR tonsillitis OR “sore throat” OR “throat infection”) AND TS=(epidemiol* OR incidence OR frequency OR rate OR surveillance OR prevalence OR burden) NOT TS=(postoperat* OR post-operat*).

APPENDIX 2: DEFINITIONS

We classified studies of children as those with participants who were predominately 18 years and younger (allowing for studies that included “school children,” for example, where a small proportion of those attending school were older than 18 years). Active surveillance was defined as studies that included a systematic and ongoing process of engaging with participants to identify episodes of sore throat. In contrast, passive surveillance was defined as studies that recorded participants with sore throat or Strep A sore throat who presented to surveillance staff during the study period or self-reported episodes retrospectively for a defined period.

Incidence was classified as either an incidence rate (IR) or a cumulative incidence. IRs were calculated as the number of episodes of sore throat or Strep A sore throat per 100 persons per year, whereas studies reporting cumulative incidence calculated the number of people with one or more episodes of sore throat or Strep A sore throat occurring during the follow-up period per 100 participants.

APPENDIX 3: JBI GLOBAL RISK OF BIAS ASSESSMENT FOR USE IN SYSTEMATIC REVIEWS

Study and year	JBI Critical appraisal checklist for studies reporting prevalence data									
	Appropriate sample frame	Appropriate sampling of participants	Adequate sample size	Study subjects and setting adequately described	Data analysis conducted with sufficient coverage of sample	Valid methods for identification of condition	Condition measured in standard, reliable way	Appropriate statistical analysis	Adequate response rate	Total score
Anwar, 2017	+	?	–	+	–	+	?	?	+	4
Casas, 2015	+	?	?	–	?	–	–	–	?	1
Danchin, 2007	+	+	+	+	+	+	+	+	+	9
DeWeyer, 2017	+	?	+	–	?	+	+	–	+	5
Di Perro, 2016	+	+	?	–	+	+	+	+	+	7
Dierksen, 2000	+	?	?	–	?	+	+	?	–	3
Hannaford, 2005	+	+	?	?	+	–	–	+	+	5
Hussein, 2005	+	+	+	+	?	–	–	+	+	6
Jose, 2018	?	?	?	?	?	+	+	+	?	3
Kamtsiuris, 2007	+	+	?	?	?	–	+	+	+	5
Karevold, 2006	+	+	?	+	?	–	?	+	+	5
Kostic, 2019	+	?	?	–	?	+	+	?	+	4
Kumar, 2009	+	?	?	–	?	+	+	+	+	5
Kumar, 2012	+	–	?	–	?	+	+	+	+	5
Kvaerner, 2000	+	+	+	+	?	–	?	+	+	6
Lennon, 2020	+	+	+	–	?	+	+	+	+	7
Lewnard, 2020	+	+	?	+	+	+	+	+	+	8
Lin, 2008	+	+	?	?	+	+	+	+	+	7
Mayxay, 2013	+	+	+	+	+	+	+	?	+	8
McDonald, 2006	+	+	?	+	?	+	+	–	+	6
McDonald, 2007	+	+	?	+	?	+	+	–	+	6
Musuku, 2017	+	+	+	+	+	–	+	?	?	6

Nandi, 2001	+	+	?	+	+	+	+	–	+	7
Negi, 2018	+	+	+	+	?	–	+	+	?	6
Parthasarathy, 2002	+	+	+	+	?	+	+	+	?	7
Steer, 2009	+	+	?	+	?	+	+	+	?	6
The score of each risk of bias domain for the listed studies by their respective symbol, with low risk (plus sign), unclear risk or not applicable (question mark), or high risk (dash).										

APPENDIX 4: CUMULATIVE INCIDENCE

Eleven studies reported episodes of sore throat, with or without tonsillitis: eight studies were predominately in children, two studies were in adults, and one study included all ages (Appendix 5). Of the eight studies in children, five reported sore throats in school-aged children (ranging from 5 to 29 years old), with a pooled cumulative incidence of 31.9 per 100 children having one or more episodes of sore throat over study periods ranging between 9 and 12 months (Figure A1)

Figure A1 Cumulative incidence of sore throat among children

Study	Country	Cases	Children	Estimate	95%-CI
Karevold, 2006 (14)	Norway	737	3406	21.6	[20.3; 23.1]
Musuku, 2017 (36)	Zambia	835	3462	24.1	[22.7; 25.6]
Hussein, 2005 (20)	Sudan	1287	3862	33.3	[31.8; 34.8]
Dierksen, 2000* (28)	New Zealand	174	490	35.5	[31.3; 39.9]
Steer, 2009* (19)	Fiji	326	667	48.9	[45.0; 52.7]
**Random effects model**				**31.9**	**[24.2; 40.8]**

Heterogeneity: $I^2 = 99\%$, $\tau^2 = 0.1895$, $p < 0.01$

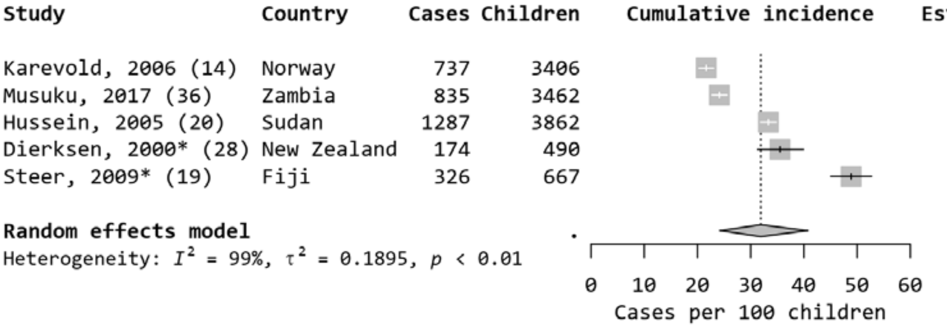

*Study duration was 10 months for Dierksen (2000) and nine months for Steer (2009).

Of the remaining three studies among children, one study reported (parent-reported) cumulative incidence among young children (4–5 years of age), finding that 6.9 per 100 children had one or more episodes over 12 months [19]. The other two studies reported the cumulative incidence of tonsillitis. The first tonsillitis study was based on retrospective reports from parents in Spain, the Netherlands, and Finland, and found that 16.1, 11.0, and 3.6 per 100 children, respectively, had experienced one or more episodes of tonsillitis during the previous 12-month period [28]. The other tonsillitis study was a nationally representative sample of 0–17 year-old children from Germany and reported that 18.5 per 100 children had one or more episodes of tonsillitis during the prior 12 months [24].

A prospective study conducted in Pakistan by Anwar et al. (2017) and a retrospective study conducted in Scotland by Hannaford et al. (2005) reported the cumulative incidence for adults. Anwar et al. reported a cumulative incidence of 56.3 per 100 persons for adults aged 18–61 years based on daily self-reporting of symptoms in the 30 days prior [27], and Hannaford et al. reported a

cumulative incidence of 30.6 per 100 adults based on patient recall of episodes of sore throat in the previous 12 months [16].

The study by Mayxay et al. (2013) from the Lao People's Democratic Republic (Lao PDR) reported the cumulative incidence from 14 randomly selected villages representing peri-urban and rural provinces. The cumulative incidence of sore throat across all ages was 2.7 per 100 persons for the previous 30 days [35].

APPENDIX 5: CHARACTERISTICS OF STUDIES WITH DATA ON THE CUMULATIVE INCIDENCE OF SORE THROAT OR TONSILLITIS

**Source**	**Study period**	**Condition under surveillance**	**Country**	**Setting**	**Study design**	**Age (in years)**	**No. of participants**	**Follow-up time (months)**	**Cumulative incidence (per 100 participants)**
Anwar, 2017 [27]	December 2012 to February 2013	Sore throat	Pakistan	Community	Prospective; patient-reported	18–61	151	3	56.3
Casas, 2015 [28]	Not specified	Tonsillitis	Spain, Netherlands, Finland	School	Retrospective; parent-reported	6–12	2,690 (Spain)<br>2,490 (Netherlands)<br>3,922 (Finland)	12	16.1 (Spain)<br>11.0 (Netherlands)<br>3.6 (Finland)
Dierksen, 2000 [30]	February 1997 to November 1997	Pharyngitis	New Zealand	School	Prospective; student-reported	5–13	490	10	35.5
Hannaford, 2005 [16]	August 1998 to October 1998	Sore throat + tonsillitis	Scotland	Community	Retrospective; patient-reported	≥14	15,788	12	30.6
Hussein, 2005 [23]	2000–2001	Sore throat	Sudan	School	Retrospective; student/parent-reported with audit of school clinical records	6–15	3,862	12	33.3
Kamtsiuris, 2007 [24]	2003–2006	Tonsillitis	Germany	Household	Retrospective; patient- (>11 years) or parent-reported	0–17	17,641	12	18.5
Karevold, 2006 [17]	2001	Tonsillo-pharyngitis	Norway	Community	Retrospective; patient-reported	10	3,406	12	21.6

Kvaerner, 2000 [19]	November 1996 to December 1996	Tonsillo-pharyngitis	Norway	Community	Retrospective; parent-reported	4–5	3,754	12	6.9
Mayxay, 2013 [35]	March 2009 to April 2009	Sore throat	Laos	Community	Retrospective; head of household-reported	All ages	9,114	1	2.7
Musuku, 2017 [38]	September 2014 to November 2015	Sore throat	Zambia	School	Retrospective; student-reported and parent-reported	5–29*<br>5–29*	3,462<br>3,462	12<br>12	24.1<br>19.0
Steer, 2009 [22]	March 2006 to November 2006	Sore throat	Fiji	School	Prospective; student self-reported	5–14	667	9	48.9

Cumulative incidence, percent of cohort with one or more episodes of disease within the study duration.

* Most (98%) participants <20 years old; n = 15 > 19 years.
